# Factors Associated With Short-Term Eradication of Rectal Colonization by KPC-2 Producing *Klebsiella pneumoniae* in an Outbreak Setting

**DOI:** 10.3389/fmicb.2021.630826

**Published:** 2021-02-01

**Authors:** Martina Pellicé, Olga Rodríguez-Núñez, Verónica Rico, Daiana Agüero, Laura Morata, Celia Cardozo, Pedro Puerta-Alcalde, Carolina Garcia-Vidal, Elisa Rubio, Mariana J. Fernandez-Pittol, Andrea Vergara, Cristina Pitart, Francesc Marco, Gemina Santana, Laura Rodríguez-Serna, Ana Vilella, Ester López, Alex Soriano, Jose Antonio Martínez, Ana Del Rio

**Affiliations:** ^1^Service of Infectious Diseases, Hospital Clínic de Barcelona, Barcelona, Spain; ^2^Service of Microbiology, Hospital Clínic de Barcelona, Barcelona, Spain; ^3^Service of Preventive Medicine, Hospital Clínic de Barcelona, Barcelona, Spain; ^4^Service of Pharmacy, Hospital Clínic de Barcelona, Barcelona, Spain

**Keywords:** decolonization, probiotic, non-absorbable antibiotic regimen, KPC-2 producing *Klebsiella pneumoniae*, outbreak

## Abstract

**Background:** KPC-producing *Klebsiella pneumoniae* (KPCKP) is a threat for patients admitted to healthcare institutions.

**Objectives:** To assess the efficacy of several decolonization strategies for KPCKP rectal carriage.

**Methods:** Observational study performed in a 750-bed university center from July to October 2018 on the efficacy of a 10-day non-absorbable oral antibiotic (NAA) regimen (colistin 10 mg/ml, amikacin 8 mg/ml, and nystatin 30 mg/ml, 10 ml/6 h) vs. the same regimen followed by a probiotic (Vivomixx®) for 20 days in adult patients with KPCKP rectal colonization acquired during an outbreak.

**Results:** Seventy-three patients colonized by KPCKP were included, of which 21 (29%) did not receive any treatment and 52 (71.2%) received NAA either alone (*n* = 26, 35.6%) or followed by a probiotic (*n* = 26, 35.6%). Eradication was observed in 56 (76.7%) patients and the only variable significantly associated with it was not receiving systemic antibiotics after diagnosis of rectal carriage [22/24 (91.6%) vs. 34/49 (69.3%), *p* = 0.04]. Eradication in patients receiving NAA plus probiotic was numerically but not significantly higher than that of controls [23/26 (88.4%) vs. 15/21 (71.4%), *p* = 0.14] and of those receiving only NAA (OR = 3.4, 95% CI = 0.78–14.7, *p* = 0.09).

**Conclusion:** In an outbreak setting, rectal carriage of KPCKP persisted after a mean of 36 days in about one quarter of patients. The only factor associated with eradication was not receiving systemic antibiotic after diagnosis. A 10-day course of NAA had no impact on eradication. Probiotics after NAA may increase the decolonization rate, hence deserving further study.

## Introduction

Infections caused by KPC-2 producing *Klebsiella pneumoniae* (KPCKP) are an increasing threat for patients admitted to healthcare institutions (McConville et al., 2017). According to the World Health Organization (WHO), multidrug-resistant microorganisms, including third-generation cephalosporin-resistant *Enterobacterales* (3GCephRE), carbapenem-resistant *Enterobacterales* (CRE), *Acinetobacter baumannii*, and *Pseudomonas aeruginosa*, are a critical priority for new antibiotic research and development (Tacconelli et al., 2018). Hand hygiene is the most effective single measure to prevent patient-to-patient transmission of multidrug resistant Gram-negative bacteria (MDR-GNB); cleaning and disinfection of surfaces, contact precautions and decolonization also contribute to prevention (Tacconelli et al., 2014).

Decolonization therapy, defined as any measure that leads to loss of detectable MDR-GNB carriage at any site, has a very low level of evidence due to the inconsistent results of the studies assessing it. In this context, a European guideline with recommendations for decolonizing regimens targeting MDR-GNB carriers in all settings was published in 2019 (Tacconelli et al., 2019). After reviewing 11 articles, the panel made a conditional recommendation against routine decolonization of CRE carriers. Nevertheless, on the basis of the limited evidence of its efficacy in high-risk patients, (Saidel-Odes et al., 2012; Oren et al., 2013) further good-quality clinical studies are needed in order to define more precisely the infection risk in populations such as colonized hematological patients and solid organ transplant recipients. These studies should set the basis for designing clinical decolonization trials.

In addition to non-absorbable antibiotics, other decolonization strategies deserve consideration. Giving the key role of dysbiosis on the loss of colonization resistance, microbiome-modifying therapies are worth exploring. These include fecal microbiota transplantation (FMT), probiotics, and prebiotics, and a range of therapies in between (Catho and Huttner, 2019; Feehan and Garcia-Diaz, 2020). In regards to probiotics, few and relatively small clinical trials have been so far unable to show any effect in preventing the acquisition of MDR microorganisms during traveling (Dall et al., 2019) or in promoting decolonization in carrier patients (Kwon et al., 2015; Salomão et al., 2016; Ljungquist et al., 2020). In addition, there is still some concern that manipulation of the microbiome could lead to unintended consequences including infections, particularly in immunosuppressed individuals and the critically ill.

An outbreak of KPCKP occurred during the summer 2018 in our hospital and, at some point during its evolution, the infection control team decided to include a decolonization regimen as part of the multifaceted control strategy. The study aim was to assess the factors associated with short-term eradication of rectal carriage with special emphasis on the role of a non-absorbable oral antibiotic (NAA) regimen and a probiotic.

## Materials and Methods

### Setting, Study Design, and Patients

This was an observational study conducted in a 750-bed university hospital in Barcelona, Spain from July to October 2018 and carried out in the context of a KPCKP outbreak.

During the outbreak period, active screening by rectal swabbing for KPCKP was done on admission and weekly thereafter to all patients admitted to high-risk wards until no new cases were detected for at least 2 consecutive weeks. All patients with a positive rectal swab for KPCKP were subjected to contact precautions and enrolled progressively into three different interventions according to the availability of a poly-antibiotic oral solution made at the hospital’s pharmacy and a commercial probiotic, as follows:

- Control group: The control group was composed of colonized patients who did not receive decolonization treatment (DT) as they were discharged before the beginning of the intervention.
- Non-absorbable antibiotic regimen (NAA): Patients in this group received 10 ml every 6 h of a mixture of colistin sulfate 10 mg/ml plus amikacin 8 mg/ml plus nystatin 30 mg/ml for 10 days.
- NAA plus probiotic regimen: The third group received the above mentioned NAA regimen followed by a probiotic (Vivomixx®) once a day for 20 days. Vivomixx® contains a combination of four Lactobacillus (*Lactobacillus paracasei* DSM 24733, *Lactobacillus acidophilus* DSM 24735, *Lactobacillus delbrueckii* ssp. *bulgaricus* DSM 24734, and *Lactobacillus plantarum* DSM 24730), three Bifidobacteria (*Bifidobacterium breve* DSM 24732, *Bifidobacterium longum* DSM 24736, and *Bifidobacterium infantis* DSM 24737), and *Streptococcus thermophilus* DSM 24731 at a concentration of 450 billion live lyophilized bacteria per sachet.

Non-absorbable oral antibiotic treatment started immediately after colonization detection and the probiotic was initiated 24 h after the last dose of NAA. Day 0 was considered the day when NAA regimen was completed (in decolonized patients) or the day of first carriage detection (in non-decolonized patients). All patients were followed up for at least 1 month. A control rectal swab was performed 1 month after day 0 in the three groups.

Patients younger than 18 years, HIV infected and those with solid and hematopoietic stem cell transplantation (HSCT) did not receive the probiotic strategy due to safety concerns.

The study was approved by the Hospital Ethics Committee (HCB/2020/0704). Written informed consent for the non-absorbable antibiotic regimen was waived because it was considered routine care for the purpose of controlling the outbreak in agreement with the policy endorsed by the Hospital Infection Control Team. Informed consent was signed by patients taking the probiotic.

### Clinical Variables, Definitions, and Outcomes

The following variables were collected from each patient: age, sex, chronic underlying diseases, chronic disease severity according to the Charlson index, performance status according to Barthel index, use of concomitant systemic antibiotic therapy during the intervention period, development of KPCKP infection, decolonization regimen, adverse reactions to the decolonization regimen and mortality.

Intestinal colonization was defined as the isolation from a rectal swab of a KPCKP strain phenotypically identical to the epidemic clone. Eradication was defined as the absence of KPCKP in the 1-month control rectal swab. Clinical infections were defined according to the CDC criteria. Antibiotics were considered active against KPCKP when the strain was susceptible according to the antibiogram. The main outcome variable was the eradication rate.

### Microbiology

Rectal swabs were processed at the local Microbiology department laboratory. They were plated on MacConkey agar (Becton Dickinson, Heidelberg, Germany) with an Aztreonam disk (30 μg), chromID ESBL agar (bioMérieux, Marcy l’Etoile, France), and chromID CARBAsmart (bioMérieux). Colonies grown on ESBL and CARBAsmart agars were identified using MALDI-TOF MS (matrix-assisted laser desorption/ionization time-of-flight mass spectrometry; Bruker, Bremen, Germany). Antibiotic Susceptibility testing was done by disk diffusion and broth microdilution methods using the Phoenix System (Becton Dickinson). The results were interpreted according to EUCAST guidelines (EUCAST 8.0, 2018). Carbapenemase production was confirmed using an immunochromatographic assay (NG-test CARBA5; NG Biotech, Guirpuy, France) from grown colonies detecting KPC, NDM, VIM, IMP, and OXA-like carbapenemases. Characterization of blaKPC gene was performed by PCR, followed by DNA sequencing.

### Statistical Methods

Groups of interest were compared with a univariate analysis by Chi-squared test, Fisher’s exact test, or ANOVA as appropriate. Those variables with a *p*-value lower than 0.2 in the bivariate analysis were introduced in a multivariate logistic regression model to determine the factors independently associated with KPCKP eradication. A value of *p* < 0.05 was considered significant. Analyses were performed using IBM SPSS Statistics version 24.

## Results

There were 93 patients involved in the KPCKP outbreak. KPC carbapenemase production was detected using lateral flow assay and the presence of *bla*KPC-2 was confirmed by PCR and sequencing (Yigit et al., 2008). The complete microbiological study of the outbreak strains is under review prior to publication.

**Figure 1:**
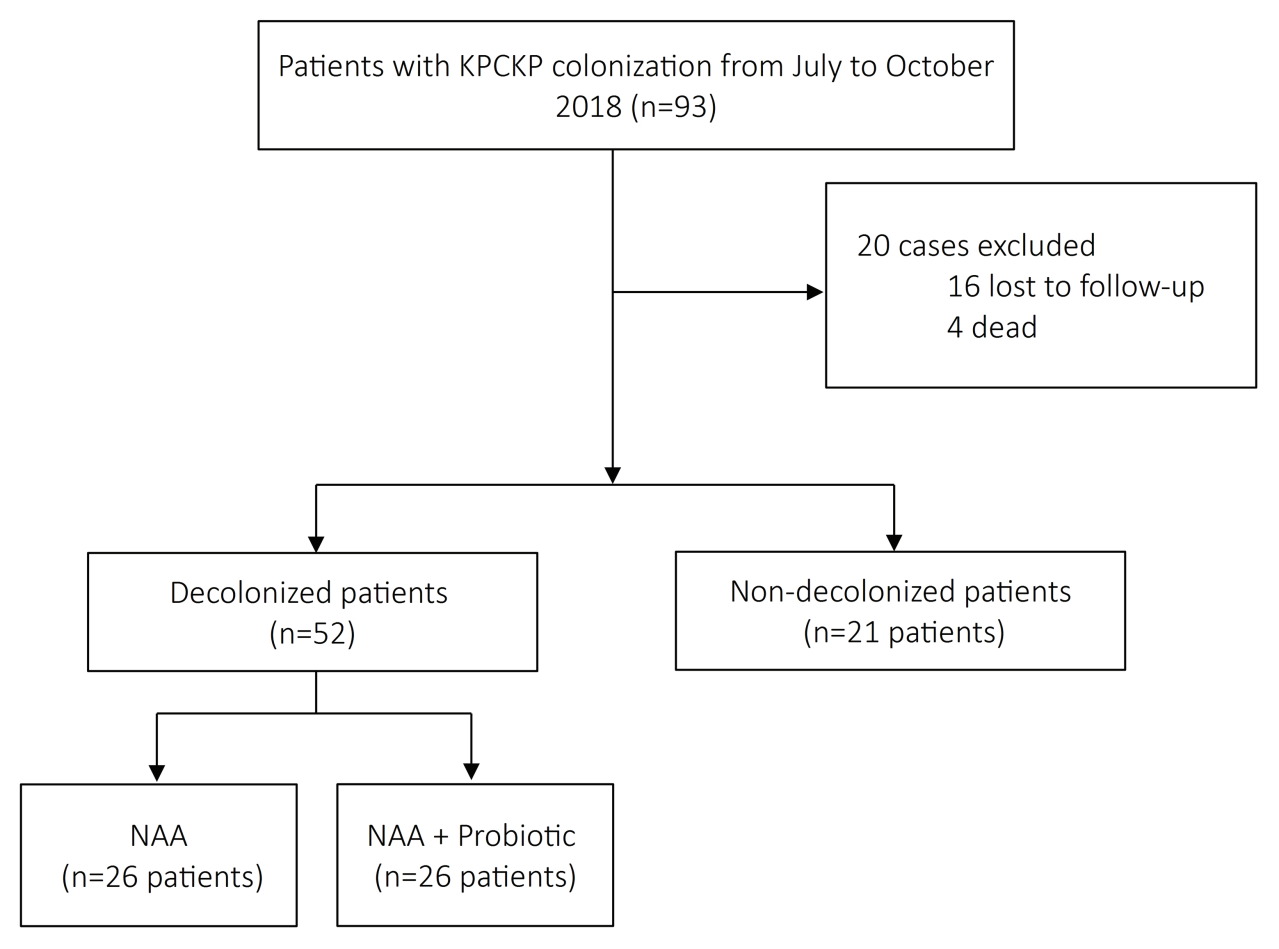
Flow chart of patients included in the study.

A total of 73 patients had a control rectal swab 1 month after completion of NAA regimen or first carriage detection and were included in the study: 21 in the control group, 26 in the NAA group, and 26 in the NAA plus probiotic group (Figure 1). Four patients died before having the control swab taken and were excluded (one in the control group, two in the NAA, and one in the probiotic group). Table 1 shows the clinical features of the patients. There were significant differences among groups in the frequency of patients with solid and HSCT as they were excluded from the NAA plus probiotic group (*p* = 0.022 and 0.039, respectively). Time elapsed from day 0 to 1-month control rectal swab was 37 ± 12 days in the control group and 41 ± 21 in the other two groups.

**Table 1:** Characteristics of patients colonized by KPCKP according to the intervention group.

Characteristics	No treatment group (*n* = 21)	NAA group (*n* = 26)	NAA plus probiotic group (*n* = 26)	*p*
Age (mean ± SD)	65.6 ± 10.2	57.3 ± 15.6	64.2 ± 14.3	0.085^a^
Female sex, n (%)	13 (62%)	15 (58%)	12 (46%)	0.52
Age-adjusted Charlson index, median (IQI)	5 (3.5–8.5)	4 (3–7)	5 (2.5–6)	0.34^b^
Barthel index, median (IQI)	100	100	100	0.30^b^
HSCT, n (%)	0	4 (15%)	0	0.022
Solid organ transplantation, n (%)	3 (14%)	6 (23%)	0	0.039
Receipt of systemic antibiotics, n (%)	12 (57%)	21 (81%)	16 (61%)	0.173

NAA, non-absorbable oral antibiotic; IQI, interquartile interval; and HSCT, hematopoietic stem cell transplantation.

a ANOVA.

b Kruskal-Wallis.

In the whole series, eradication rate at 1 month of follow-up was 76.7% (56 patients). Factors associated with eradication are shown in Table 2. A significant association was observed between receiving systemic antibiotics after diagnosis of rectal carriage and lack of eradication (the 1-month control rectal swab was negative in 34 (69.3%) of 49 patients taking antibiotics vs. 22 (91.6%) of 24 patients not receiving them (OR = 0.2, 95% CI = 0.042–0.99, *p* = 0.04).

**Table 2:** Univariate analysis of factors associated with eradication.

Characteristic	Eradication (*n* = 56)	No eradication (*n* = 17)	OR (95% IC)	*p*
Age, mean ± SD	61.8 ± 13.8	63.4 ± 15.1	-	0.67
Female sex	30 (54%)	10 (59%)	0.8 (0.3–1.9)	0.7
Age-adjusted Charlson index, median (IQI)	5 (3–7)	4 (2–7.5)	-	0.40
Barthel index, median (IQI)	100	100	-	0.81
HSCT, n (%)	3 (5%)	1 (6%)	0.9 (0.16–5.3)	0.93
Solid organ transplantation, n (%)	9 (16%)	0	-	0.078
Receipt of systemic antibiotics, n (%)	34 (61%)	15 (88%)	0.2 (0.042–0.99)	0.041
Decolonization treatment, n (%) - None - NAA - NAA plus probiotic	15 (27%) 18 (32%) 23 (41%)	6 (35%) 8 (47%) 3 (18%)	Reference 0.9 (0.25–3.1) 3.0 (0.6–14)	0.8 0.14

IQI, interquartile interval; HSCT, hematopoietic stem cell transplantation; and NAA, non-absorbable oral antibiotic.

Although the intervention group was not significantly associated with the primary outcome, the rate of decolonization at 1-month in patients receiving NAA plus probiotic was numerically higher than that of controls [23/26 (88.4%) vs. 15/21 (71.4%), OR = 3, 95% CI = 0.66–14, *p* = 0,16] and of those receiving NAA only (OR = 3.4, 95% CI = 0.78–14.7, *p* = 0.09).

An exploratory multivariate logistic regression analysis retained two variables in the model: receiving systemic antibiotics (OR = 0.14, 95% CI = 0.03–0.73, *p* = 0.02) and the interaction of the NAA plus probiotic group with the administration of systemic antibiotics (OR = 4.3, 95% CI = 0.8–25, *p* = 0.086). This interaction reflects the fact that taking NAA plus probiotic was almost significantly associated with a higher eradication rate in the subgroup of patients that received systemic antibiotics (14/16, 87% vs. 20/33, 61%; OR = 4.5, 95% CI = 0.9–23, *p* = 0.055), however, this trend was not observed in patients not receiving systemic antibiotics (9/10, 90% vs. 13/14, 93%; OR = 0.69, 95% CI = 0.04–12.5, *p* = 1).

Clinical infections were diagnosed in 13 (17.8%) patients. In nine of them, the infection was present on detection of rectal carriage and in four it appeared more than 48 h later. Isolates proceeded from urine culture in seven patients; blood cultures in three, and ascitic fluid, osteoarticular tissue, and endometrial aspirate in one each. After detection of rectal carriage, no clinical infections were observed in the control group, three (11.5%) were found in the NAA group (urinary tract infection, spontaneous bacterial peritonitis, and bloodstream infection) and one (3.8%) in the NAA plus probiotic group (urinary tract infection). These last four patients achieved eradication in the 1-month control swab.

Some patients complained of the bad taste of the NAA solution, but not other adverse events were reported. Both the 17 KPCKP strains isolated from the 1-month control rectal swab and the four from the clinical samples remained susceptible to colistin and amikacin.

## Discussion

The results of this observational study suggest that in terms of KPCKP eradication from stools after 1 month of detection or after finishing a 10-day NAA course, taking a NAA solution consisting of colistin and amikacin was not better than not taking it. However, there is also some suggestion that in comparison with no treatment or NAA alone, the administration of a probiotic after the NAA regimen may increase the 1-month decolonization rate. In addition, this study indicates that the main factor deterring eradication is exposure to systemic antibiotics after carriage detection, and that the beneficial effect of the probiotic-containing regimen seems to be limited to the subgroup of patients exposed to them.

Failure of NAA to achieve stable eradication of multiple-drug resistant Gram-negative bacilli in general or KPCKN in particular has been previously described (Saidel-Odes et al., 2012; Lübbert et al., 2013; Bar-Yoseph et al., 2016). For KPCKP decolonization, the most commonly used regimens have been based on colistin (1 vs. 2 MU four times daily) and/or gentamicin (80 mg four times daily). The clinical guidelines of ESCMID-EUCIC suggest designing decolonization assays with oral colistin sulfate (50 mg four times daily) with or without gentamicin sulfate (80 mg four times daily; Tacconelli et al., 2019). However, this recommendation may be questionable. Some authors suggest that 100 mg of colistin sulfate (about 67 mg of colistin base or 2 MU) every 6 h may be the most effective dosage (Silvestri et al., 2013). Previous studies on neutropenic patients had suggested that when colistin was given alone, 800 mg/day was perhaps the most appropriate regimen (Clasener et al., 1987). It is of note that inactivation of colistin in feces (90–95%) is significantly reduced when co-administered with tobramycin and this was the rationale for combining aminoglycosides with lower colistin dosages (Clasener et al., 1987). The NAA solution used in the present study contained 2 MU per dose and yet, we did not see any effect *per se* on the eradication rate. The reason for the accompanying aminoglycoside being amikacin is that this oral solution was designed as the decolonizing regimen for selected high-risk patients carrying MDR-GNB with high rates of resistance to gentamicin and tobramycin.

In the present study, the lack of efficacy of NAA alone was mainly explained by the high rate of 1-month spontaneous eradication. The meta-analysis performed by Bar-Yoseph et al. (2016) included six and five studies for a total of 239 and 172 patients to assess the rate of CRE persistent carriage among healthcare residents at 3 and 12 months, respectively. The carriage decreased from 74.6% at 3 months to 34.6% at 12 months. Feldman et al. (2013) followed 125 KPCKP carriers monthly for 3 months. Spontaneous decolonization was observed in 48% at 1 month and in 52% at 2 months. However, in the persistent carriers, 70% remained positive at 6 months (Feldman et al., 2013). In our study, we observed a spontaneous decolonization rate of 71% (15/21), which was higher than that reported by others. We do not have a satisfactory explanation for these discrepancies. The absence of clinical infection in this group may suggest a lower bowel burden than in the other groups.

The lack of NAA efficacy in the present study does not automatically dismiss a given decolonization strategy as clinically useless. Even without achieving stable eradication, NAA can transitorily decrease the load of the colonizing microorganisms below the threshold burden for clinical infection or effective transmission. Oral decontamination with aminoglycosides has been associated with a lower risk of infections and mortality in high-risk patients colonized by colistin-resistant KPCKP (Machuca et al., 2016). In our study, the risk of clinical infection was low and did not seem to be significantly influenced by the decolonization strategy. This supports the recommendation that only patients with a well-defined high risk of infection should be the focus of any decolonization therapy.

In this study, the complementation of the NAA with a probiotic increased the eradication rate (from 33/47, 70.2% in the control or NAA groups to 23/26, 88.4% in the NAA plus probiotic group, OR = 3.25, 95% CI = 0.8–13, *p* = 0.07), but almost exclusively in patients receiving systemic antibiotics after carriage diagnosis. Over the last 10 years there has been an increasing public and scientific interest in the administration of probiotics to prevent or treat diseases. Regarding MDR-BGN decolonization, clinical evidence is still limited. A randomized study in 103 ICU patients published in 2015 did not find significant differences in acquisition or loss of MDR organisms including *Pseudomonas aeruginosa* and ESBL or CRE, between the group of patients that received *Lactobacillus rhamnosus* GG (one capsule containing 10^10^ CFU every 12 h for 14 days) and the one that did not (Kwon et al., 2015). In another randomized trial, the administration of a symbiotic (10^10^ CFU *Lactobacillus bulgaricus* plus 10^10^ CFU *L. rhamnosus* plus fructo-oligosaccharides twice a day for 1 week) or placebo to 101 evaluable hospitalized patients colonized by MDR-GNB was not associated with improved rates of decolonization or infection (16.7% in the treated group and 20.7% in the control group, *p* = 0.60; Salomão et al., 2016). Lastly, in a more recently published randomized trial, Vivomixx® (the same product used in the present study) or placebo were administered to 80 ambulatory patients colonized by ESBL-*Enterobacterales* (mainly *Escherichia coli*) with poor results (12.5% eradication rate with the probiotic vs. 5% with placebo, *p* = 0.24; Ljungquist et al., 2020). Regarding KPCKP, a small pilot trial has been mentioned in an Italian journal in which 36 elderly frail hospitalized patients were randomized to receive high-dose probiotic and psyllium for 14 days or standard care. The authors reported a significant reduction in carriage rate in the probiotic group during hospitalization (53 vs. 12%, *p* = 0.009; Nouvenne et al., 2015).

We found that exposure to systemic antibiotics was the main factor associated with no eradication. This is not unexpected for antibiotics with poor *in vitro* activity against KPCKP or for *in vitro* active agents achieving low concentrations in the intestinal lumen. Systemic antibiotics probably perpetuate dysbiosis and increase the bacterial burden of resistant microorganisms (Wang et al., 2017), making them even less eradicable by NAA. Most remarkable was our observation that the probiotic increased the eradication rate almost exclusively in patients taking systemic antibiotics after the diagnosis of carriage. It is perhaps in this unfavorable scenario for eradication, where NAA regimens are insufficient and could be improved with additional interventions directed to restore the integrity of the gut with a healthy colonic microbiota (De Rosa et al., 2015).

Development of resistance to the components of the decolonization regimen is of particular concern. Machuca et al. (2016) found that a significantly higher proportion of patients undergoing decolonization had gentamicin-resistant isolates in follow-up cultures than those not treated (13 vs. 3%; *p* = 0.008). In the Lübbert et al. (2013) and Oren et al. (2013) studies, 14% (7/50) and 28% (4/14) of patients, respectively, developed secondary resistance to decolonizing agents. Emergence of resistance may be more frequent if monotherapy is used (Oostdijk et al., 2013). In our experience, the KPCKP strain was susceptible to both colistin and amikacin and, as reported by others (Saidel-Odes et al., 2012), no resistance to these antibiotics developed.

The most important limitations of the study are its observational design, the relatively low number of patients, its unicentric characteristic and the short follow-up with eradication defined as only one negative swab at 1 month. In addition, the exclusion from administering the probiotic to solid organ and hematopoietic stem cell transplantation patients added heterogeneity to the intervention groups. Lastly, neither quantitative studies to measure the impact of the interventions on fecal KPCKP load nor microbiota analysis were done.

## Conclusion

In fecal carriers of KPCKP, decolonization with a 10-day NAA regimen based on colistin and amikacin was not effective for increasing the eradication rate at 1 month of follow-up. However, the administration of a probiotic for 20 days after the conclusion of the NAA regimen was associated with a numerically higher eradication rate, particularly in patients who received systemic antibiotics after diagnosis of rectal carriage. No effect on the rate of clinical infections was noted. The present study supports current recommendations against the systematic use of NAA in patients colonized by MDR-GNB, which should be focused on populations with documented high risk of infection. In our opinion, further studies are needed to examine the efficacy of giving probiotics after a NAA regimen, particularly in colonized patients who have to receive systemic antibiotics.

## Data Availability Statement

The raw data supporting the conclusions of this article will be made available by the authors, without undue reservation.

## Ethics Statement

The studies involving human participants were reviewed and approved by Hospital Ethics Committee (HCB/2020/0704), Hospital Clínic de Barcelona. The patients/participants provided their written informed consent to participate in this study.

## Author Contributions

MP, JM, AS, and AR were responsible for the study design. MP, OR-N, VR, DA, LM, CC, PP-A, CG-V, ER, MF-P, AVe, CP, FM, GS, LR-S, AVi, EL, AS, JM, and AR were involved in the collection and analysis of samples to implement the study. MP, JM, and AR performed the analysis and interpretation of the data. MP and JM had a major contribution in writing the manuscript. All authors contributed to the article and approved the submitted version.

### Conflict of Interest

The authors declare that the research was conducted in the absence of any commercial or financial relationships that could be construed as a potential conflict of interest.
